# Assessing the effectiveness of Australian early childhood education and care experiences: study protocol

**DOI:** 10.1186/s12889-016-2985-1

**Published:** 2016-04-21

**Authors:** Collette Tayler, Daniel Cloney, Ray Adams, Karin Ishimine, Karen Thorpe, Thi Kim Cuc Nguyen

**Affiliations:** The University of Melbourne, Victoria, Australia; Australian Council for Educational Research, Melbourne, Australia; Queensland University of Technology, Brisbane, Queensland Australia

**Keywords:** Early education and care, ECEC, Longitudinal tracking, Quality programs, E4Kids, Program effectiveness, Socio-economic status, Families, Home learning environment, Children’s wellbeing

## Abstract

**Background:**

In Australia, 61.5 % of children aged 3–4 attend Early Childhood Education and Care (ECEC) programs. Children’s experiences within these programs vary widely and impact directly on educational wellbeing and social development. Research has shown that higher quality programs enhance children’s learning and developmental outcomes, foster social participation and have long-lasting effects on their productivity as adults. Quality matters, yet we do not know what components of ECEC result in a quality program.

Effective Early Educational Experiences (E4Kids) is a 5-year longitudinal study designed to identify and assess the impact of mainstream ECEC programs and program components on children’s learning, development, social inclusion and well-being. E4Kids sets out to measure quality ECEC; identify components that add value and positively impact children’s outcomes; evaluate the effects of child, family, community and environment characteristics on programs; and provide evidence on how best to invest in ECEC.

**Methods/design:**

E4Kids follows a sample of 2,494 children who have experienced a variety of approved care programs (long day care, kindergarten, family day care and occasional care), as well as 157 children who have not accessed such programs. Children are tracked to the first point of National Assessment Program – Literacy and Numeracy (NAPLAN) testing at Year 3. The study presents a multi-level design in which ECEC programs were sampled from two states – Queensland and Victoria – then randomly sampled from two greater metropolitan regions and two regional and remote locations.

Parents, centre directors, educators and carers complete questionnaires to provide information on demographics and children’s progress. Data collected also include the make-up and organisation of ECEC programs and schools children attended. The quality of adult-child interactions is directly assessed using the Classroom Assessment Scoring System (CLASS) and direct testing of children’s cognitive abilities and achievements is undertaken over 3 years and linked with NAPLAN scores.

**Discussion:**

Findings from the E4Kids study have the potential to influence the quality of ECEC available in Australia by providing up-to-date evidence on the impact of ECEC programs and program components to inform future policy decisions and research.

## Background

Converging evidence from developmental and economic science identifies the first years of life as a sensitive developmental period in which life-long social participation and productivity are established [1–3]. From conception to age five, significant brain development and neural structuring occurs [1]. The experiences children have at this time determine whether a child’s developing brain architecture provides a strong or weak foundation for future learning, behaviour and health [3]. Early learning experiences establish the pathways for children’s motivation for school learning and long-term scholastic attainment [1, 3, 4] and children’s early interactions and relationships with adults and peers establish pathways for their emotional security, sense of agency, self-regulation and social behaviour [1].

Economic analyses [5, 6] suggest that the early years offer the greatest return to investment in human capital because: (1) positive life trajectories are established in the early years and (2) the need for more costly, less effective remediation can be averted [2]. Randomised control trials of early intervention, conducted in the US, demonstrate this causal relationship. In these studies, children who received preschool education and were supported by their parents achieved greater life success (e.g. college completion, higher earnings) and experienced less adversity (e.g. need for special education, participation in crime, welfare support) by age 40, than comparable children who did not attend a program. Moreover, cost-benefit analyses applied to longitudinal data from these studies indicate that the return on investment in the ECEC programs was as high as US$17 for each dollar originally spent [2]. Thus, early experiences count and optimising early experiences through effective ECEC programs is a policy option with potential to benefit individuals and society.

### Study context

In Australia, children enter school with widely different preparation for their ongoing learning and social participation [1, 2]. These differences reflect diverse early experiences that have already played a major role in establishing children’s life prospects [3]. While the home environment is the primary source of experience for young children [7], 76 % of Australian 3–4 year olds take part in non-parental early childhood education and care (ECEC) programs [8]. The experiences children have within these programs vary widely and impact their learning and developmental outcomes. High quality programs increase children’s life chances through to adulthood and have the greatest effects on disadvantaged children [2, 4]. In contrast, the absence of child participation in an ECEC program is a predictor of poor progress [2, 4, 9], with lower quality programs resulting in short-term effects [4], no effect [1], or even negative effects [1] on children’s outcomes in the early years of school.

Clearly, quality ECEC provision is important. However, understanding of what constitutes quality provision in Australia, and the value obtained from the $8.6 [10] billion annual investment in ECEC by Australian governments, is limited. There is a need to understand the effect of attending a program (or not doing so), the relative effect of different programs, and their constituent parts, in promoting children’s learning, social well-being and on-going life chances. This study asks: Are Australian ECEC programs effective? Which are most effective? In what ways are these programs effective? For whom are they effective? And for how long do the effects endure?

### Rationale for the study

The Effective Early Educational Experiences (E4Kids) study investigates the effectiveness of ECEC programs in Australia. It was designed immediately before and conducted during a national ECEC sector reform agenda targeted to “ensure that by 2020 all children have the best start in life to create a better future for themselves and for the nation” [11]. This meant that the E4Kids study was situated at a time and place when the contribution of ECEC programs to children’s learning and development is in sharp focus. The reform agenda includes policy initiatives pertaining to non-parental ECEC that focus on the years immediately prior to preschool (at 3–4 years) to build the quality of existing ECEC provision and enhance access. E4Kids inherently aligns to this policy direction through its design; E4Kids seeks to examine the delivery of ECEC programs at age 3–4 years, and is positioned to inform on-going investment in ECEC policy and research.

Empirical evidence similarly verifies the E4Kids focus; reporting that programs attended by children aged 3–4 affect attainment at school entry and have enduring effects on children’s outcomes at primary school [4, 9]. ECEC programs prepare children for social participation and learning at school. Although family background and early experiences within the family are an important component for explaining some of these differences, so too are children’s experiences in ECEC programs. The issue is, however, that not all ECEC program types are equally effective in establishing the foundations for social participation and learning [4]. What program elements deliver stronger and more enduring effects on child outcomes? This question defines the quality that E4Kids seeks to explore.

The E4Kids study seeks identify and define quality ECEC and its effects on children’s outcomes in Australia. It aims to provide evidence about ECEC programs in a diverse range of Australian communities, including remote, regional and urban locations, and incorporates Indigenous and disadvantaged children as an imperative focus.

The study identifies key components of quality within and across the subsidised ECEC program types, including long day care (LDC), family day care (FDC), kindergarten (K) and occasional (or limited hours) day care (ODC). The contributions of each of these different programs to children’s learning and developmental outcomes is tracked across a 5-year period and may be compared to a no program control (NPC) group of children. In 2010, a large cohort of 3–4 year olds was selected and their on-going learning and development was monitored to the first wave of national testing, in Year 3, at age 8.

The assessment of the contribution of ECEC programs to child outcomes captures both the long reach of ECEC intervention through longitudinal design and the wide reach in measuring a diverse range of outcomes. Selection of outcome measures has been guided by the Council of Australian Governments (COAG) productivity agenda [12] to include important indicators on learning and development. Findings from this study will inform theory on ECEC provision and will have implications for policy, investment and practices relating to ECEC provision in Australia.

### Aims

1. To identify and define quality in ECEC by measuring and assessing the independent contributions of program scope, structure and pedagogical practices;
2. To evaluate the independent effects of ECEC programs, at 3–4 years, on children’s learning, cognitive and social development, social inclusion and well-being, by controlling for family background, family learning environment, prior non-parental care, and community;
3. To evaluate the independent effects of ECEC programs on family participation, social inclusion and well-being, controlling for family background, family learning environment, prior non-parental care, and community;
4. To evaluate investment in ECEC programs by understanding the contribution of program components that add value to child outcomes and to assess, through comparison of relative effectiveness, the returns on those investments to children, families and the community.

## Methods/design

### Framework

The study adopts an ecological theoretical framework [13], which asserts that a child’s developmental attainments and well-being are embedded within the contexts of the family, the ECEC program and the broader social and economic community. A key feature of the design is that it positions the evaluation of ECEC programs within diverse communities, across Victoria and Queensland, selected on the basis of both advantage and their “risk” to children’s outcomes [14] (population characteristics) and program access (location). Children not attending ECEC programs were selected as a no-program control [NPC], and their care environments and outcomes were measured. Concurrent economic data will enable accurate analyses of on-going investment effectiveness supported by longitudinal cost-benefit analyses.

### Sampling methodology

To address the key research aims of the E4Kids study, a cluster-randomised sampling design was used to select a cohort of children attending typical or ‘everyday’ ECEC programs. The cohort was recruited in 2010 and participated longitudinally until child-records data linkage in 2015. The process used to achieve the final sample involved identifying: (1) the target population (for which the results from E4Kids intend to be generalised), (2) the sampling frame that represents that target population (the achieved population), (3) the target sample, and (4) the achieved sample. This approach to sampling is based on other large education studies [15, 16].

E4Kids focuses specifically on children participating in approved^1^ ECEC programs in Australia. Therefore, the target population included a subset of children participating in ECEC programs in Australia. This is an important distinction for E4Kids since other large Australian data sets include more general information about children and their development. The scope of the target population was reduced by three contextual factors: population density constraints, child age constraints and funding and access constraints. Population density constraints reduced the scope of the target population because very remote areas of Australia have low population densities and no access to everyday ECEC provision. Some very remote areas receive other forms of provision, including mobile or visiting services, while others receive no provision at all [17]. Areas that did not provide typical everyday ECEC programs were excluded from the target population.

Child age constraints reduced the scope because of the variability of the ages of children participating in different forms of ECEC provision. To normalise the age ranges of children from different provision types and ensure all major provision types were included in the study, the target population was reduced in scope to include children who participated in ECEC classrooms that usually included 3–4 year old children. By implication, this excluded, for example, infant classrooms in long day care services.

Funding and access constraints reduced the scope of the target population by limiting the total size of the study. Yet to maintain the integrity of estimates and achieve generalisable results, a sufficient number of classroom-child observations needed to be made. Since the study was part-funded by the State Government jurisdictions of Queensland and Victoria, the target population was limited to within these states. To maximise the available budget, minimise the need for travel between sites, and to produce a sample that was representative of the diversity within Australia, regions were deliberately selected as the study’s sites including the Statistical Divisions of major metropolitan Queensland and Victoria (metropolitan); and the Statistical Local Areas of a greater regional area in Victoria (regional) and a remote location in Queensland (remote).

The achieved population was sourced from regulatory lists of licensed ECEC programs in the four study regions. These lists – provided by the State Government partners and current for the year 2009 – comprised the sampling frame. The sampling frame was explicitly stratified by location (metropolitan, regional, and remote) and service type (LDC, K, FDC, ODC). Some minor forms of ECEC services were excluded (Early Childhood Inclusion Services and Restricted Licenses in Victoria, representing less than 1 % of all programs) as per the scope of the sample design. This yielded 16 explicit strata.

A target sample of 150 services and 2,500 children was set based on the likely range of the design effect, to ensure that sample estimates would be generalisable. This target sample was split proportionally between each stratum to establish the target numbers of ECEC services within each stratum presented in Table 1. Within each explicit stratum, implicit stratification was used to ensure a spread of services from high and low SES (Socio-Economic Status) neighbourhoods. Each stratum was weighted by neighbourhood SES and service capacity, to ensure that ECEC services in the highest and lowest quartiles of SES would be included in the sampling process.

**Table 1:** Description of achieved population and target sample by sampling stratum

cs	Metropolitan Queensland				Metropolitan Victoria				Regional Victoria				Remote Queensland			
	LDC^a^	K^b^	FDC^c,d^	ODC^e^	LDC^a^	K^b^	FDC^c,d^	ODC^e^	LDC^a^	K^b^	FDC^c,d^	ODC^e^	LDC^a^	K^b^	FDC^c,d^	ODC^e^
Achieved population size	733	143	30	10	870	795	37	296	12	25	2	13	5	4	1	0
Average capacity	63	33	35	22	68	35	44	22	77	31	120	23	66	23	22	.
Total capacity	46242	4735	1055	195	58731	27425	1635	6654	927	763	240	301	331	93	22	.
Target Sample size	56	11	2	1	30	14	3	3	8	7	1	4	5	4	1	0
Sampling interval	825.8	430.5	527.5	195.0	1957.7	1958.9	545.0	2218.0	115.9	109.0	240.0	75.3	66.2	23.3	22.0	.
Total replacements	21	7	0	1	8	6	1	1	0	1	0	2	0	0	0	.
Minimum replacement order^f^	0	0	0	1	0	0	0	1	0	0	0	0	0	0	0	.
Maximum replacement order^f^	4	2	0	1	3	2	3	1	0	1	0	2	0	0	0	.

^a^Long Day Care

^b^Stand-alone, typically sessional kindergarten programs for children in the year before school and 3-year-old programs in Victoria

^c^Family Day Care

^d^Service capacity was not available in 2009 licensing data. Data reported are the number of FDC schemes in each stratum (achieved population size and target sample size) and the number of FDC carers within each stratum (capacity). FDC homes usually included between 4 and 7 children

^e^Occasional day care or limited hours care

^f^Replacement order is the number of services selected to replace the originally samples service. Zero reflects that that the service originally sampled agreed to participate

Stage one of sampling occurred from September to December 2009 and involved the random selection of ECEC services of proportional size (as measured by the total licensed capacity in each stratum) from the sampling frame. Within the sampling frame, with services now listed and weighted by neighbourhood area SES, a new vector was created to represent the weighted cumulative sum of the capacity in each stratum and ranged from one to the sum of the weighted capacity for each stratum. A random number within the range of the cumulative sum vector was drawn to comprise the first service sampled. The remaining target number of services was sampled by going down the list of ECEC provider names using a pre-determined sampling interval, and looping back into the top of the sampling frame when the bottom of the list was reached.

Information letters were sent, and follow-up phone calls were employed to each selected ECEC service provider to explain the study and to invite the director of the ECEC service to participate. Services that did not agree to participate where replaced by the service that was listed next on the sampling frame. If the replacement service did not agree to participate either, then the next replacement was the service listed above the originally sampled service in the sampling frame. This ‘nearest neighbour’ replacement strategy was used until a service that was similar to the first sampled service agreed to participate. Table 1 shows that a minimal total replacement sampling was conducted in the study. However, when replacement sampling was required, it was usually the next service listed in the sampling frame that agreed to participate.

Stage two of sampling was conducted in the first quarter of 2010. It involved recruiting clusters of children, aged three and four, from classrooms in the services (that agreed to participate) in stage one. Each of these services was audited using a standardised schedule that listed all possible characteristics of an ECEC classroom – for example, the type, capacity, and an age-range of all classrooms in each service were recorded. Classrooms that included five or more children between the ages of three and four were included in E4Kids and all children in selected classrooms were invited to participate. In FDC situations, households were recruited if they included as least one child aged between three and four.

This process of sampling achieved a sample of 2,494 children, drawn from 142 recruited services, for E4Kids. The longitudinal nature of the design meant that the services and classrooms included in subsequent years were non-randomly selected; as participating children progressed into preschool and school classrooms selected by their families, these services and classrooms were consequentially recruited into the study. In 2011 and 2012, 721 and 806 ECEC and schools services, respectively, participated. Within these ECEC and school programs, there were a total of 286; 1,136; and 1,427 classrooms in 2010, 2011, and 2012, respectively. The study continued in 2013, 2014 and 2015, including data linkage with school sector evidence on children’s progress and performance. A summary of the achieved sample is given in Tables 2 and 3.

**Table 2:** Achieved E4Kids sample of ECEC and school services and children within them

	FDC^a^	K^b^	LDC^c^	S^d^	O^e^	U^f,g^	Total
Services 2010	7	40	92	0	2	1	142
Children 2010	100	818	1542	0	27	7	2494
Services 2011	8	186	153	371	2	1	721
Children 2011	51	629	720	932	12	150	2494
Services 2012	3	70	53	677	1	2	806
Children 2012	10	100	109	1923	2	350	2494

^a^Family Day Care

^b^Stand-alone, typically sessional kindergarten programs for children in the year before school and three-year-old programs in Victoria

^c^Long Day Care

^d^School

^e^Other, including limited hours and occasional care programs

^f^For Classroom rows, count of rooms where either Room or Service type data is missing

^g^For Children rows, count of children who were allocated to a classroom or where either Room or Service type data is missing

**Table 3:** Achieved E4Kids sample of ECEC and school classrooms and children within them

	FDC^a^	K^b^	LDC^c^	KLDC^d^	S^e^	O^f^	U^g,h^	Total
Classrooms 2010	59	71	93	21	0	2	40	286
Children 2010	100	741	1106	224	0	27	296	2494
Classrooms 2011	35	299	143	71	576	9	3	1136
Children 2011	51	631	549	167	931	12	153	2494
Classrooms 2012	9	88	42	31	1253	2	2	1427
Children 2012	10	101	61	47	1922	2	351	2494

^a^Family Day Care

^b^Stand-alone, typically sessional kindergarten programs for children in the year before school and three-year-old programs in Victoria

^c^Long Day Care

^d^K programs provided in LDC settings

^e^School

^f^Other, including limited hours and occasional day care programs

^g^For Classroom rows: count of rooms where either Room or Service type data is missing

^h^For Children rows: count of children who were allocated to a classroom or where either Room or Service type data is missing

The achieved sample was split approximately equally by gender: 1,199 females (48 %), 1,294 males (52 %) and 1 non-response. Children’s ages at January 1 in each year of the study are shown in Table 4, and reinforces the diversity in ages when recruiting children participating in everyday ECEC programs in Australia that include children aged three to four.

**Table 4:** Child ages at 1 January in each year of the study

Year	*n*	*M*	*SD*	min	max
2010	2493	42.92	7.27	12	68
2011	2493	54.92	7.27	24	80
2012	2493	66.92	7.27	36	92

### Sampling of the No Program Control (NPC) group

Children who did not attend a program were the NPC group. The best approximation for children not in approved care was the residual of a list of families receiving subsidy for approved care subtracted from a list of all families known to have children of a given target age. No single department held both pieces of data: the Department of Education, Employment and Workplace Relations (DEEWR) held records of the families who received subsidy for care in the Child Care Management System (CCMS) and the Department of Families, Housing, Community Services and Indigenous Affairs (FaHCSIA) held records of families with children of given ages in the Family Tax Benefit administrative records. From the residual group, it was necessary to subtract those families who used kindergarten programs (funded by state government) and those who did not use subsidies but used approved care. Consultation with FaHCSIA and DEEWR suggested that the recruitment rate of the target sample could be as low as 3 %. Therefore, it was decided to deliberately oversample to offset an overly low recruitment rate and ensure a reasonable achieved sample size.

The NPC sample was explicitly stratified by location and age to mirror the E4Kids sample. Nine-hundred families were selected and stratified: 346 each from the greater urban area in each state, and 104 each from regional Victoria and remote Queensland. In addition, the children from families needed to fit within the following age ranges, in recognition of the entry conventions in each State: in Queensland, children should be born after June 30, 2006 and before June 29, 2007; in Victoria, children should be born after April 30, 2007 and before April 29, 2008.

Nine-hundred families represented 4.32 % of the sampling frame (*N* = 20,826), including 7.4 % of the urban Queensland sampling frame (*N* = 4,661), 2.2 % of the urban Victorian sampling frame (*N* = 15,668), 29.4 % of the regional Victorian sampling frame (*N* = 353) and 72.2 % of the remote Queensland sampling frame (*N* = 144).

After a two-week opt-out period, a two-staged recruitment process was undertaken. Initially, all sampled families were sent an E4Kids recruitment pack that included a statement about the study, a consent form and a short survey asking about their use of ECEC services to screen out any families whose children had previously participated in approved programs. Following this, all families who had not returned a consent form and screener, were phoned. When contact could not be made, a message was left (where possible) and families were followed up a maximum of three times (at different times or days, unless instructed otherwise) over a 2-week period. A final mail-out was conducted to all remaining families that had not been reached.

A screening tool was used to identify families who utilised kindergarten programs or approved programs but did not receive government subsidy; however, families remained eligible if they used any amount of informal care, including playgroups. Families were screened out if:

1. They used approved care or kindergarten for more than 10 h per week in a *typical* week, unless they had used these programs for less than 3 months in 2010.
2. The child fell outside the nominated age range.

Of the 900 NPC families sampled, 59 opted out via the FaHCSIA phone line. The greatest barrier to recruitment was contacting families: 364 (43 %) of the 841 families sampled were unable to be reached. Of the remaining 477 families, 322 (67.5 %) either declined to participate in the study, were screened out because of ECEC use or age ineligibility or did not return a consent form. One-hundred fifty-five families (32.5 %)^2^ agreed to participate and were recruited to comprise the NPC.

### Weighting methodology

The methodology used in E4Kids to calculate sampling weights reflects the best standards of practice and aligns with international studies of educational achievement [15, 16]. Sampling weights were calculated for all children and services recruited in 2010. Services in subsequent years were recruited non-randomly (i.e. as a consequence of children moving into and through services, as mentioned previously). Therefore, in the cross-sectional years (2011 and 2012) services were equally weighted.

The **service weight** was interpreted as the number of services that each sampled service represented in the population. The weight of a service (*i)* was denoted, *W*_*i*_. For remote Queensland all services were selected with certainty and therefore *W*_*i*_ equaled one^f^. In all other strata, the weight of a service was calculated as the product of a base weight, a correction factor and a trimming factor, as shown in Eq. 1. Where a service was selected and replaced by another service, the participating service inherited the weight of the originally sampled service.

Equation 1: Service weight function.

1 $$ {\boldsymbol{W}}_{\boldsymbol{i}}={\boldsymbol{w}}_{\boldsymbol{i}}{\boldsymbol{f}}_{\boldsymbol{i}}{\boldsymbol{t}}_{\mathbf{1}\boldsymbol{i}} $$

Where *w*_*i*_ is the base weight of a service *i* that (approximately) sums across selected services in a stratum, to give the total number of services in the stratum, and is given by Eq. 2 below.

Equation 2: Service base weight function.

2 $$ {w}_i=\frac{int\left(\frac{{\displaystyle \sum }mos}{n}\right)}{mo{s}_{i\ }} where\ int\left(\frac{{\displaystyle \sum }mos}{n}\right)>mo{s}_{i\ }\  else\ 1 $$

$int\left(\frac{{\displaystyle \sum }mos}{n}\right)$ is the sampling interval within the explicit stratum, given by the sum (within the stratum) of measures of size (the capacity of each service), divided by the number of services within the stratum.

One service, in regional Victoria, had *mos*_*i*_ greater than the sampling interval, and received a base weight of 1 as per the conditional statement in Eq. 2. Thus the sum of selected service base weights was an approximation of the count of services within the stratum, with some random perturbations due to chance (e.g. start value and sampling intervals < *mos*_*i*_).

*f*_*i*_ was a correction factor to account for implicit oversampling of services in high and low SES communities. During sampling, services were ordered by the SES of the community they were a part of, as measured by the Socio-Economic Index for Areas (SEIFA) Index of Relative Socio-Economic Advantage and Disadvantage (IRSAD), and were then randomly selected proportionally to size (random start, selecting every service that includes the *j*^*th*^ student – the sampling interval). The first and fourth quartiles, or IRSAD, within each stratum, were weighted greater than the middle quartiles in the proportions 35, 15, 15, 35. The correction factor was therefore 0.25/0.35 for services in the first and fourth quartiles, and 0.25/0.15 for services in the middle quartiles.

*t*_1*i*_ was a trimming factor to reduce the weights of services with very large values of *w*_*i*_. Large values of *w*_*i*_ occurred when services with very small *mos*_*i*_ relative to other services in the stratum, were selected; they received very large base weights because of their low probability of selection. To compensate for this, the mean value for *mos*, *M*(*mos*), within the stratum was calculated, and services with *mos*_*i*_ ≤ *M*(*mos*)/1.5 inherited a trimming factor equal to less than one, which reduced their influence on parameter estimates. Services with *mos*_*i*_ > *M*(*mos*)/1.5 inherited a trimming factor equal to one. The trimming factor for services with a capacity less than 1.5 of the *M*(*mos*) were given by the ratio of the *w*_*i*_’(the service base weight), with *M*(*mos*) replacing *mos*_*i*_. Therefore, the trimming factor can never be greater than one. Fifteen per cent of services in the sampling frame received a value for the trimming factor not equal to one. The formula for *t*_1*i*_ is further explained by Eqs. 3 and 4.

Equation 3: Calculation of service weight prime for services with small measure of size.

3 $$ {w}_i\hbox{'}=\frac{int\left(\frac{{\displaystyle \sum }mos}{n}\right)}{\mathrm{M}(mos)} where\ mo{s}_i\ \le M(mos)/1.5\  else\ {w}_i $$

Equation 4: Function for service trimming factor.

4 $$ {t}_{1i} = \frac{w_i\hbox{'}}{w_i} $$

When calculated, the mean service weight in the achieved sample was 16.28 (*SD* = 15.59, min = 0.77, max = 72.62). Weights had a minimal impact on parameter estimates. Note that weighted estimates were given by non-parametric empirical bootstrap using 500 replications in the boot library of R [18].

### Measures

Table 5 presents a detailed summary of the measures and items selected for E4Kids, with corresponding explanations for the variables. Participating children were tested at least three times on standard outcome measures. Direct measures of the children included:

**Table 5:** Summary of constructs measured

Level	Domain	Methods	Timeline	Variable details
Child	Demographics	Parent Questionnaire	April 2010–2014 each year	Place of birth, age, gender, ethnicity, cultural identity, birth order.
	Health	Parent Questionnaire	April 2010–2014 each year	Weight at birth, weeks of pregnancy, birth situation, multiple birth, breastfed, food and nutrition, health condition, history of disability and mental status.
	Education/Care	Parent Questionnaire	April 2010–2014 each year	ECEC program starting age, program type/s, duration, reasons, cost, history of education/care, Social Learning Environment (SLE) Questionnaire, Short Temperament Scale for Children (STSC), Strengths and Difficulties Questionnaire (SDQ).
Family	Demographics	Parent Questionnaire	April 2010–2014 each year	Marital status, relationship, family composition, age, employment status, Household Demographic Questionnaire, income, occupation, education, ethnicity, mobility (Contact Details Questionnaire).
	Health	Parent Questionnaire	April 2010–2014 each year	Caregiver Health Questionnaire, Family Functioning Questionnaire, Family Well-Being Questionnaire, Family mental health (K10).
	Education/Care	Parent Questionnaire	April 2010–2014 each year	Parenting, home language, Home Learning Environment (HLE) Questionnaire, engagement with ECEC program and community.
	Social	Parent Questionnaire	April 2010–2014 each year	Social Support Questionnaire.
Program (Centre/room-level)	Scope	Director Questionnaire	May 2010	Screener (director’s name & contact person’s name, address, email address, number of “target age” rooms/groups).
				Centre characteristics (name, primary service, hours of operation, opening/closing times, physical setting, legal status (for-profit/for-non-profit/government).
				Enrolment (total number of children in age category, licensed capacity, a waiting list, children with special needs, additional support for children with special needs).
				Program & Facilities (centre philosophy, services from outside sources, services for parents, centre space (indoor & outdoor)).
				Staff Resources (number of grouping, group age category, staff employment status, staff qualification, role of the staff/teacher in group, professional development, centre fees, staff wages, standard wages at the centre, percentage of salary budget).
				Target ages room (number of enrolled by gender, number of staff, staff gender, staff age range, role of each staff, staff employment status, staff contact hours with children, qualifications, number of practicum students, number of volunteer, daily schedule, children with special needs, cultural background, children with non-English speaking background, language support, programming time).
	Quality indicator 1	Director Questionnaire	May 2010	Staff qualification, staff composition, staff diversity, role of staff, staff professional development, contact staff turnover, staff-child ratios, group size, parent program.
		Early Childhood Environment Rating Scale – Revised (ECERS-R)	July - September 2010	
				Classroom observation.
				Three subscales – Space and furnishings, Personal care routines, Activities.
	Quality indicator 2	Classroom Assessment Scoring System (CLASS)	July - September 2010 (1 WAVE)	Classroom observation (20 min x 4/6).
				Three domains – Emotional Support (positive climate, negative climate, teacher sensitivity, regard for student perspectives), Classroom Organization (behaviour management, productivity, instructional learning support), and Instructional Support (concept development, quality of feedback, language modelling).
			2011 (2 WAVES)	
			2012 (1 WAVE)	
			2013 (1 WAVE applied to a sub-sample of 100 scoop classrooms in Queensland)	
	Economics	Director Questionnaire	May 2010	Program costs ([1] total program costs including labour, infrastructure, staff etc. and [2] costs per child).
		Data Linkage with DEEWR & FaHCSIA		Program financing ([1] private contribution including fees, volunteers, donations etc. [2] public contribution including direct fee subsidy (e.g. demand side fee subsidies: CCB, CCTR) and indirect fee subsidy (including grants, exemptions etc.).
Program (Staff-level)	Scope	Staff Questionnaire	June 2010	Staff demography (name, gender, age range, cultural background, education, employment status, student status, number of years’ experience, prior experience).
				About the room (number of enrolment by gender, number of staff, staff gender, staff age range, role of each staff, staff employment status, staff contact hours with children, qualification, number of practicum student, number of volunteer, daily schedule, children with special needs, cultural background, children with non-English speaking background, language support, programming time).
				Curriculum (philosophical approach, links to EYLF/VEYLDF (VIC), emphasis on academic program, pedagogical methods (whole-group vs small group), learning strategy (teacher-led vs child-initiated), assessment, outside source of program, goals of curriculum, program evaluation).
				Professional Development (availability of professional development, duration, content, support from the centre)
				Staff work review, Reason’s for working in ECEC, Staff attitude and beliefs.
Children	Physical	Anthropometry	April to June 2010	Height, Weight, Waist circumference.
	Cognitive	Woodcock Johnson (WJ-III)	April to June 2010	Cognitive: Test 1 Verbal Ability.
				Cognitive: Test 5: Concept Formation.
				Cognitive: Test 6: Visual Matching.
	Achievement	WJ-III	April to June	Achievement: Test 4: Understanding Directions.
			2010	Achievement: Test 10: Applied Problems
			2011	Cognitive: Test 18: Rapid Picture Naming
			2012	Achievement: Test 21: Sound awareness.
	Social Inclusion	Bus Story	April to June 2010	Children work with researchers to select the adventure and build a story. Stickers “☺” are used by the children to nominate 3 friends to take on the bus with them. Each child completes their bus individually and is interviewed by a researcher about the reasons for their nomination.
	Adult-Child Interaction	Thorpe Interaction Measure (TIM)	April to June 2010	The stimulus is a novel picture book with photographs representing children engaged in a number of activities. Each book has 10 stimulus photos.

- Cognition and achievement of individual children: Woodcock Johnson III (WJ-III; established standardised assessment tool) used each year, commencing April 2010.
- Measurement of height, weight and waist circumference: recorded at each wave of data collection to identify children’s physical growth.
- National numeracy and literacy scores (NAPLAN) in Year 3: obtained by data linkage from State Government partners.

In addition, the interaction amongst children (social inclusion and friendship) was measured using ‘Bus Story’ (a participation exercise; 2010 and 2011). However, the Bus Story tool was not used for the NPC. A parent survey was delivered to parents of participating children to gather family-related information. Adult-child interaction measures included:

- Observational assessments of the quality of adult-child interactions in ECEC: Classroom Assessment Scoring System (CLASS).
- Observation of adult-child interactions based on picture-story telling in ECEC: Thorpe Interaction Measure (TIM), applied in 2010 and 2011.

ECEC services information included:

- Space and furnishings, personal care routines and activities in ECEC: Early Childhood Environmental Rating Scale – Revised.
- Teacher/Educator survey (for educators working directly with the children).
- Program Director or School Principal survey (included specific questions relating to the cost of approved care for the purpose of economic analysis).
- Audit of the attendance of children in the programs.

### Researcher training

More than 40 research assistants were employed each year to undertake data collection. Training on WJ-III, Bus Story and TIM was conducted over two full days. It comprised group training at the university and implementation piloting of each measure on children in LDC centres. The researchers assessed the children and submitted scored test booklets for feedback and verification of appropriate scoring. A further three day training program on the CLASS and ECERS-R was conducted each year, followed by clinical reliability testing of all researchers. Researchers who did not meet the reliable performance criteria (>80 % fidelity on all observed items) did not proceed to collect data.

### Analytic strategy

**Fig. 1:**
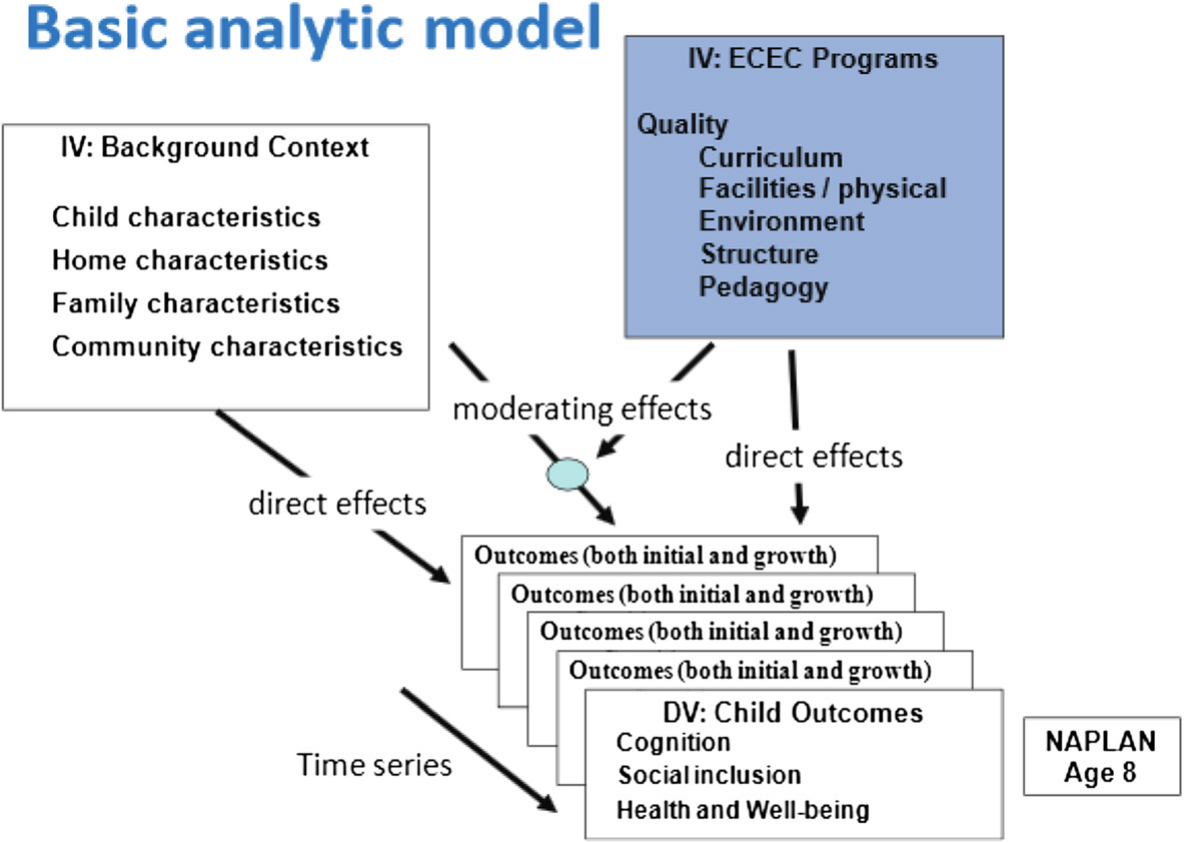
E4Kids analytic model

The study design was developed for multi-level modelling in which child, family, program and community levels of influence on children’s outcomes will be analysed. The basic analytic model for E4Kids is presented in Fig. 1. Currently, analyses of the E4Kids data are underway, and will address the research questions of the study in the following manner:

1. What features of ECEC provision promote children’s learning and social participation and define quality? Here analyses will focus on the comparison of data from Wave 1 (baseline) and Wave 2, and comparison of data from Wave 1 and Wave 3. Analyses will control for community, family and prior ECEC history, to compare child outcomes across and within program types. This will enable examination of the features of programs that best predict children’s outcomes. Features that consistently predict outcome, regardless of program type, will be identified.
2. How does the ECEC experience affect children’s on-going development, educational attainment and social well-being? Children’s outcomes at NAPLAN testing will be modelled using community, family and program-level data, and will control for prior learning at Waves 2 and 3. Modelling will identify universal and context specific predictors of success.
3. How do ECEC program inputs influence children’s developmental outcomes (educational attainment & social well-being)? Program data will act as an independent variable to identify outcomes that are significant predictors for children’s outcomes and child, family and community characteristics.

In addition, concurrent cost and price data will be analysed to enable the study to compare and contrast the change in child outcomes achieved through different programs (within and between program types). This will be achieved by using two distinct approaches that respond to the following questions:

4. How cost effective are ECEC programs? Cost Effectiveness Analysis: Cost per significant developmental gain will be contrasted between programs. A program is cost-effective if it delivers desired effects at a lower cost per unit than alternative programs. A more robust understanding of the elements of quality will allow for comparison of individual program characteristics that enhance children’s development. There is also potential to raise program effectiveness with negligible impact to cost, through building enhanced understanding of the elements of program quality.
5. What is the long-term return on investment in ECEC? Cost Benefit Analysis: It is possible to begin an analysis of ECEC programs in terms of their relative worth to the individual, public and society. Through statistical analysis, if an ongoing effect is found in achievement, then ECEC programs may play a role in deferring future remediation costs. By measuring the accrued benefits independently attributed to ECEC program participation, and contrasting them against a matched NPC, or low quality program group, a robust estimate of the net benefit to participant, family and community can be ascertained.

### Ethics

This study is conducted under the approvals and protocols sanctioned by the University of Melbourne Human Research Ethics Committee (ID 0932660.2), and in accordance with linked approvals provided by the Victorian Government Department of Education and Training, the Queensland Department of Education and Training and the relevant Catholic Education Archdioceses. In accordance with the ethical approvals, formal written consent was obtained from each study participant, including the child’s main caregiver, the educators in programs, and school and service leaders. Verbal consent to take part in, or decline, each of the assessment activities was also obtained from each participant child, and all participants maintained the right to withdraw their participation at any time.

### Availability of data and materials

In accordance with the terms and conditions agreed by the parties engaged in the study, data and study materials are owned by the University of Melbourne, and available the participating parties and researchers under license, for use in accordance with the approvals granted to the research team by the participants.

## Discussion

The E4Kids study is innovative in two key ways.

### Focus

This is the first study of the effectiveness of ECEC programs in Australia. The longitudinal design captures the long reach of quality in ECEC through to the first point of national testing at age 8, for which the Partner Organisations provide data linkage. The attention to measurement captures the wide reach of quality in ECEC. The study assesses not only the gains in educational attainment and productivity (human capital) but also social outcomes that include dimensions of health and social well-being (social capital).

### Methodology

The multi-level study design focuses on quality of provision, taking account of community, program and family contributions to child outcomes. Comparisons will be made within and across communities. The design allows examination of the effectiveness of not only program type, but also variation within program types, and contrasts with the NPC. Great attention is taken with measurement to map to currently untested developmental and economic hypotheses concerning the mechanisms by which ECEC programs promote human and social capital formation. Furthermore, development of new measures alongside typically chosen measures contributes to theorising the key constructs of human and social capital development.

E4Kids directly addresses national research priorities for promoting and maintaining good health: a healthy start to life; and strengthening Australia’s social and economic fabric. As such, the study addresses a key element of the nation’s productivity agenda [11, 12]. The E4Kids study is designed to benefit through:

1. Contribution to knowledge: The study represents a culmination of considerable research conducted by the team both nationally and internationally. This collective work deepens our current understanding of the effectiveness and costs of early education and care. Furthermore, the Australian context affords a unique opportunity to contribute to knowledge through the provision of a NPC group.
2. Contribution to policy: COAG acknowledge the importance of ECEC for the nation’s long-term prosperity and productivity. Since 2007, ECEC investment has increased but there is clear demand for evidence to inform this investment. This study addresses this need and plays a key role in informing national policy, investment strategy and practice both in ECEC and formal education.
3. Contribution to practice: The study aims to articulate quality in ECEC and focuses on a wide range of quality components: scope and access; structure; and pedagogy and curriculum. Findings have direct relevance to the teaching of young children, with the possibility of improving their life prospects.

Dissemination of the results of E4Kids in order to effectively communicate key findings to academics, policy-makers and practitioners, nationally and beyond is the primary work of the study in and beyond 2016. To date, annual reports of implementation progress and early findings have been documented via: internal reports; newsletters; culturally and language-appropriate printed materials; workshops and conferences with members of the partner organisations; and journals.

## Abbreviations

(CCMS): child care management system
(CLASS): classroom assessment scoring system
(COAG): council of australian governments
(DEEWR): department of education, employment and workplace relations
(E4Kids): effective early educational experiences
(ECEC): early childhood education and care
(FaHCSIA): department of families, housing, community services and indigenous affairs
(FDC): family day care
(IRSAD): index of relative socio-economic advantage and disadvantage
(K): kindergarten
(LDC): long day care
(NAPLAN): national assessment program – literacy and numeracy
(NPC): no program control
(ODC): occasional (or limited hours) day care
(OECD): organisation for economic co-operation and development
(SEIFA): socio-economic index for areas
(SES): socio-economic status
(TIM): Thorpe interaction measure
(WJ-III): Woodcock Johnson III assessment tool

## Footnotes

1 ‘Approved’ is a term indicating that an early childhood program is eligible for government subsidy in order to make fees more affordable for families.

2 Two of these families had two children in the target range, providing 157 children in the NPC sample NPC sample.
